# Acute, chronic, and detraining effects of dynamic stretching and tissue flossing on muscle function, sprint performance, balance, and proprioception, in soccer players with hamstring shortness: a three-arm parallel randomized controlled trial protocol

**DOI:** 10.3389/fspor.2026.1919926

**Published:** 2026-09-21

**Authors:** Mohammad Alghosi, Hassan Daneshmandi, Ali Shamsi Majelan, Andreas Konrad

**Affiliations:** 1 Department of Sports Injury and Corrective Exercise, Faculty of Physical Education and Sport Sciences, University of Guilan, Rasht, Iran; 2 Institute of Human Movement Science, Sport and Health, Graz University, Graz, Austria

**Keywords:** athlete performance, dynamic balance, jump performance, muscle power, muscle strength, range of motion, team sports, tissue flossing

## Abstract

**Objective:**

Hamstring shortness is prevalent among soccer players and is associated with an increased risk of muscle strain and performance deficits. Dynamic stretching and tissue flossing are two interventions used to address flexibility impairments; however, research has predominantly focused on their acute effects. A significant gap remains in understanding their isolated, individual chronic (≥2 weeks) and detraining effects on comprehensive athletic performance. Hence, this randomized controlled trial protocol aims to compare the acute, chronic, and detraining effects of dynamic stretching versus tissue flossing on muscle function, sprint performance, balance, and proprioception in soccer players with hamstring shortness.

**Methods:**

A three-arm, parallel randomized controlled trial will be conducted. A minimum of 57 male adolescent soccer players with clinically diagnosed hamstring shortness will be randomly assigned to a dynamic stretching group, a tissue flossing group, or a control group. The intervention groups will perform their respective protocols three times per week for six weeks. The outcomes include hamstring range of motion, muscle strength, and muscle power, 20-meter sprint time, dynamic balance, and knee joint proprioception. All outcomes will be assessed at four time points: baseline, immediately after the first session (acute), after six weeks of training (chronic), and after a three-week detraining period (9 weeks).

**Discussion:**

This trial will provide novel evidence on training- and detraining-related adaptations in response to two distinct intervention strategies. The findings are expected to clarify whether one modality offers superior immediate performance enhancement compared to chronic flexibility gains, and how well these effects are retained after detraining. The results will directly inform evidence-based recommendations for integrating these techniques into athletic training and injury prevention programs for soccer players.

**Clinical Trial Registration:**

https://irct.ir/trial/87892, identifier: IRCT20251202068197N1.

## Graphical Abstract

**Figure:**
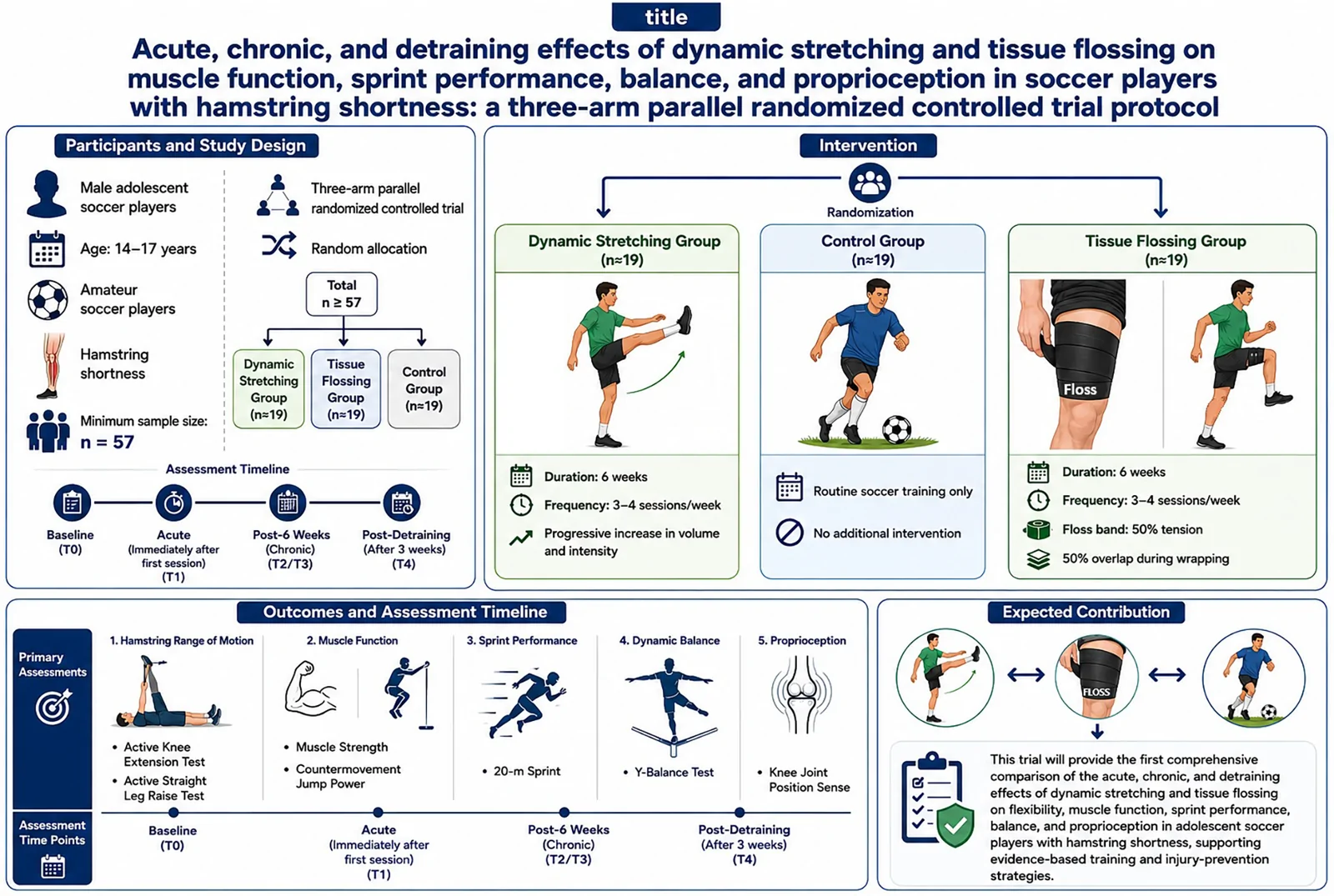

## 1 Introduction

Hamstring injuries are a major concern in soccer due to their high occurrence, particularly affecting the isolated proximal biceps femoris during high-speed movements (1). These injuries are more common towards the end of each half, possibly because hamstring strength decreases as matches progress (2). In this regard, alterations in hamstring length and flexibility lead to movement and postural disorders (3–5). Hamstring shortening impacts the lumbar spine and pelvis and elevates the risk of leg injuries stemming from muscle imbalances, ultimately causing more injuries and lost playing time (6–9). In sports like soccer, due to the increased prevalence of hamstring shortness, assessing the effectiveness of various flexibility-enhancing routines is essential, especially for young athletes, to prevent decreased flexibility and possible injuries (9–11). Interventions to counteract hamstring shortness include stretching, indirect hamstring treatment using osteopathic techniques, ultrasound-guided neuromodulation, neurodynamic exercises, muscle energy technique, massage, soft tissue mobilization, dry needling, and Nordic hamstring exercise (12–16).

While various stretching modalities [static, dynamic, ballistic, and proprioceptive neuromuscular facilitation (PNF)] enhance flexibility, research demonstrates that their effectiveness varies over time. Meta-analytic evidence suggests that, acutely, dynamic stretching (DS) produces flexibility improvements similar to those of static and PNF stretching. However, in long-term applications, DS actually shows smaller effect sizes in flexibility gains compared to static and PNF approaches (4, 8, 17, 18). However, DS has demonstrated improvements in athletic performance and may help mitigate the incidence of injuries. In contradistinction to static stretching, DS is theorized to be more physiologically and functionally aligned with the biomechanical demands of athletic activities. This renders it a potentially more effective modality for pre-participation preparation. It may be particularly beneficial in sports necessitating explosive power, sprinting, and range of motion (ROM) (8, 17, 19, 20).

In contrast to DS, tissue flossing (TF) has emerged as a popular alternative for improving flexibility and performance. Introduced by Starrett and Cordoza, the technique involves wrapping a 7-foot, 2-inch thick latex band around a limb, with the aim of enhancing range of motion, athletic performance, and recovery, while reducing pain (21). Application typically involves overlapping the band by 50% with each wrap for 1 to 3 min. Although most research has concentrated on the short-term benefits of TF, such as increases in ROM and functional performance, its long-term impact on overall athletic capabilities remains unclear, despite its growing use among athletes and therapists (22–27).

While a few studies have explored the effects of DS and TF on hamstring flexibility, a gap remains in understanding their isolated acute and chronic effects (defined as those occurring over≥2 weeks of intervention) (4, 28). Furthermore, the effects of detraining, how well gains are retained after ceasing the intervention, are poorly understood for both modalities, despite evidence that flexibility adaptations partially regress, yet substantial residual benefits are maintained post-training (29). The rationale for comparing these specific interventions stems from their distinct physiological mechanisms and practical applications. DS primarily enhances neuromuscular activation and functional ROM through active movement patterns (30–32), while TF represents a novel mechanical approach that may improve blood flow and fascial mobility through compression and subsequent reperfusion (33). Both techniques are increasingly used in athletic settings; however, their comparative efficacy across the comprehensive set of functional and neuromuscular outcomes remains unclear.

The overarching aim of this protocol will be to advance the development of optimized hamstring intervention strategies for soccer players by delineating the distinct functional outcomes associated with DS and TF. The objective is to compare the acute, chronic, and detraining effects of these two interventions on hamstring flexibility, muscle strength and power, and sprint performance, balance, and proprioception in soccer players with hamstring shortness. Given that TF applies compressive force and subsequent reperfusion, which may acutely modulate pain perception, fascial mobility, and neural drive (22), we hypothesize that TF will yield greater immediate (acute) improvements in muscle function, sprint performance, balance, and proprioception compared to DS. Furthermore, due to the distinct mechanisms of each intervention, with DS promoting active neuromuscular adaptation and TF relying on transient mechanical effects, we anticipate that the chronic and detraining effects will differ between DS and TF across these domains. Specifically, we expect DS to induce more sustained improvements in functional outcomes, while the effects of TF may be less retained following the detraining period. Ultimately, this work seeks to generate evidence-based recommendations for selecting the most effective hamstring intervention strategy, with the expectation that both interventions will demonstrate distinct profiles of efficacy across functional and neuromuscular outcomes in this athletic population.

## 2 Methods

### 2.1 Study design and ethical considerations

The study follows the CONSORT guidelines (34) and the 2013 version of the Declaration of Helsinki (35). Additionally, it is designed according to the Standardized Protocol Items: Recommendations for Interventional Trials (SPIRIT) statement (36). Ethical approval for this study was obtained from the University of Guilan Ethics Committee (IR.GUILAN.REC.1404.149) on December 1, 2025 and was registered in the Iranian Registry of Clinical Trials (IRCT20251202068197N1) on 5 December 2025.

Participant recruitment commenced in June 2026 and is currently ongoing. Data collection is in progress. The trial is expected to be completed by September 2026. This protocol is submitted for publication during the active recruitment phase, and no data analysis has been conducted at the time of submission.

A three-arm parallel randomized controlled trial will be employed in this study. Participants will be randomly assigned to one of three groups: a DS group, a TF group, or a control group. While the intervention groups will engage in DS and TF protocols, the control group will perform only routine exercises. To evaluate the effectiveness of each intervention, ROM, muscle function, sprint performance, balance, and proprioception will be assessed at four time points: pre-test (baseline), immediately after the first session (acute effect), post-test after six weeks (chronic effect), and post-intervention follow-up (after three weeks of detraining). The selected duration of three weeks is justified by evidence indicating that a training deficiency during this period can adversely affect both strength and neuromuscular performance across various athletic populations (37–39). During the 3-week detraining phase, participants will discontinue the specific intervention protocols (DS or TF) administered in the study. However, to maintain their athletic conditioning and for ethical reasons, as the study is conducted during their pre-competition active phase, all participants, including those in the control group, will continue with their standard, team-based soccer training and routines throughout this period.

To minimize bias and ensure participant understanding, a familiarization session will precede the baseline assessment, while a separate session will verify hamstring shortness. Prior to the start of this investigation, written informed consent will be obtained from the parents/guardians of all participants who are under 18 years of age. The detailed flowchart is shown in Figure 1. Also, Table 1 displays the enrollment schedule, interventions, assessments, and relevant data for the participants.

**Figure 1:**
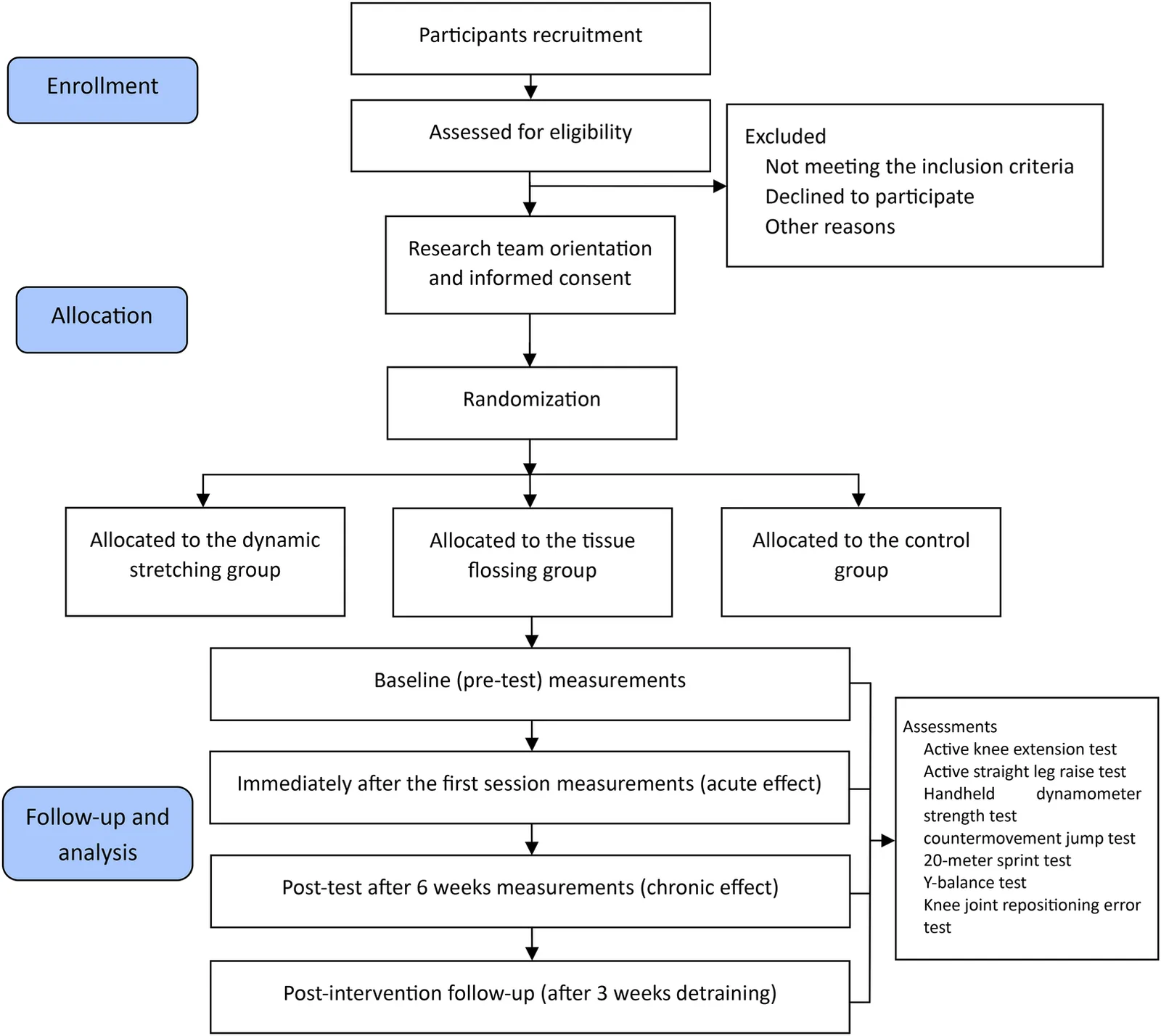
Study design flow chart.

**Table 1:** Schedule of enrolment, interventions, assessments, and data collection.

	Enrolment & Screening	Allocation & Baseline	Acute effect measurement (after the first session)	Intervention period (6 weeks of training)	Chronic effect measurement (after 6 weeks of training)	Follow-up Period (after 3 weeks of detraining)
TIMEPOINT	-t_1_	t_0_	t_1_	t_2_	t_3_	t_4_
**ENROLMENT**						
Eligibility screen	X					
Informed consent	X					
Allocation		X				
**INTERVENTIONS**						
Dynamic stretching group		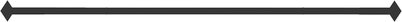				
Tissue flossing group		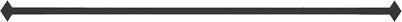				
Control group		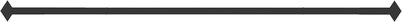				
**ASSESSMENTS**						
Active knee extension test		X	X		X	X
Active straight leg raise test		X	X		X	X
Handheld dynamometer strength test		X	X		X	X
countermovement jump test		X	X		X	X
20-meter sprint test		X	X		X	X
Y-balance test		X	X		X	X
Knee joint repositioning error		X	X		X	X

### 2.2 Study participants

The population will include male soccer players of Qazvin province. Inclusion Criteria: (1) Male amateur soccer players (who play in local leagues without a financial contract) (40) aged 14 to 17 years; (2) Minimum of 3 years of continuous soccer participation within organized soccer clubs; (3) Clinically diagnosed with hamstring shortness, defined as a score of more than 33 degrees on the active knee extension test (41); (4) No prohibition on using DS or TF, with the approval of a medical professional. Exclusion Criteria: (1) Any current or recent (within the past 6 months) lower limb musculoskeletal injury, surgery, or condition that could affect testing or training; (2) Inability to commit to the full study duration and prescribed protocols, including more than two consecutive or three total absences.

### 2.3 Recruitment and informed consent

Participants will be recruited from the population of male amateur adolescent soccer players within the Qazvin province. Recruitment strategies will include direct engagement with local soccer clubs participating in regional leagues, distributing study information through sports federations and school-based athletic programs, and running targeted advertisements on relevant social media platforms. Recruitment commenced in June 2026 and is anticipated to span approximately 4–6 weeks, depending on participant availability and scheduling. The trial is currently in the active recruitment phase. Prior to enrollment, written informed assent will be obtained from all participants, alongside written informed consent from their parent or legal guardian. The study procedures, potential risks, and benefits will be thoroughly explained.

### 2.4 Sample size

*A priori* sample size calculation was performed using G*Power 3.1 (Heinrich-Heine-Universität Düsseldorf, Germany) for a repeated-measures ANOVA, focusing on within-between interactions. The parameters were set as follows: an effect size of 0.19, a significance level (*α*) of 0.05, statistical power of 0.80, three groups, and four measurements. The effect size of f = 0.19 was derived from Yao et al. (2026) (42), a systematic review and meta-analysis of tissue flossing, which reported a pooled effect size of Hedges’ g = 0.19 (95% CI: 0.05–0.33) for range of motion, and Hedges’ g = 0.19 (95% CI: −0.03 to 0.41) for muscle strength, the two primary outcomes of this trial. Based on these inputs, the calculated sample size was 51 participants (17 per group). Anticipating a 10% attrition rate, we aim to recruit 57 participants.

### 2.5 Randomization, allocation concealment, and blinding

Eligible participants will be randomly assigned to one of three groups, including a DS group, a TF group, or a control group, using a computer-generated randomization sequence from https://www.randomizer.org. The randomization list is securely stored on a password-protected computer, accessible only to the researcher responsible for participant allocation. Recruitment staff and others will not have access to this information. Due to the study's design and nature, participants and the research personnel delivering the interventions cannot be blinded to group allocation. However, all outcome assessors and data analysts will be blinded to group assignment. Specifically:

1. Assessors conducting physical measurements will not have access to group allocation information.
2. Data analysts processing video recordings will be blinded to group allocation during all analyses.

To maintain blinding, all data files will be coded using participant identification numbers only, and the allocation key will be stored separately and securely, accessible only to the principal investigator, who is not involved in outcome assessments or data analysis.

### 2.6 Interventions

#### 2.6.1 Dynamic stretching program

All participants in the study will begin each intervention session with a standard 8-minute warm-up protocol that includes a slow jog (300 meters), high-knee exercise (300 meters), 10 squats, 30-second jumps, and a 2-minute run at 30% effort (43). After warming up, all participants in the DS group will complete the 6-week protocol bilaterally, with sessions conducted in the morning (10–12 AM) during the pre-competition active phase, following the specific frequency, intensity, time, and type parameters (30, 44, 45) detailed in Table 2.

**Table 2:** Dynamic stretching protocol (6 weeks).

Week	Frequency	Intensity	Time	Type
1-2	3 times per week	Controlled movements to the end of the range of motion, but less than the point of discomfort.	3 sets (15 reps) 3 sets (15 reps) Total time: 8 min	Active leg swing (sagittal plane) Lunge with eccentric hamstring windmills
3-4	3 times per week	Increase the range of motion slightly while maintaining control and staying below the point of discomfort.	4 sets of (20 reps) 4 sets of (20 reps) Total time: 12 min	Active leg swing (sagittal plane) Lunge with eccentric hamstring windmills
5-6	4 times per week	Increase the range of motion and speed of movements, while ensuring movements remain controlled and pain-free.	5 sets of (20 reps) 5 sets of (20 reps) Total time: 16 min	Active leg swing (sagittal plane) Lunge with eccentric hamstring windmills

Descriptions: All exercises will be performed at a moderate tempo (3 s per repetition: 2 s eccentric phase, 1 s concentric phase), synchronized to a metronome set at 60 beats per minute. A 20-second rest interval will separate sets.

#### 2.6.2 Tissue flossing program

After the same warming up, participants assigned to the TF group will undergo floss band (FB) application, with pressure maintained as described in the previous study (46). The tissue FB to be used in this study (Kintex; Apaloo GmbH, Riedweg 10, 78607 Talheim, Germany; kintex.de) will have the following specifications: 208 cm in length, 5 cm in width, and 1.5 mm in thickness, made from 100% latex. A black band will be chosen for its suitable resistance for athletic use. The band will be anchored just above the distal third of the thigh, between the anterior superior iliac spine and the patella. It will be applied in a spiral pattern (distal to proximal), maintaining 50% tension and 50% overlap between layers. The FB remains applied continuously throughout each session (no removal between sets). The protocol will be conducted in the morning (10–12 AM) during the pre-competition active phase, with exercises performed bilaterally. To ensure a standardized technique, the FB will be applied by trained research personnel. The characteristics of the TF program (47, 48) are outlined in Table 3.

**Table 3:** Tissue flossing protocol (6 weeks).

Week	Frequency	Intensity	Time	Type
1-2	3 times per week	The band is applied with 50% tension and 50% overlap between layers.	2 sets (15 reps) 2 sets (15 reps) Total time: 8 min	Active leg swing (sagittal plane) Active standing toe touches with a slight knee bend
3-4	3 times per week	The band is applied with 50% tension and 50% overlap between layers.	3 sets of (20 reps) 3 sets of (20 reps) Total time: 12 min	Active leg swing (sagittal plane) Dynamic standing hamstring stretch with elevated foot
5-6	4 times per week	The band is applied with 50% tension and 50% overlap between layers.	4 sets of (20 reps) 4 sets of (20 reps) Total time: 16 min	Active leg swing (sagittal plane) Standing hamstring scoop with reach

Descriptions: All exercises will be performed at a moderate tempo (3 s per repetition: 2 s eccentric phase, 1 s concentric phase), synchronized to a metronome set at 60 beats per minute. A 20-second rest interval will separate sets.

#### 2.6.3 Control group program

Participants in the control group will not receive DS of FB applications during the study period. After completing the same warm-ups, they will engage in a light, standardized active control activity lasting the equivalent duration (8–16 min) as the intervention protocols. This activity will consist of low-intensity ball familiarization (e.g., light dribbling, simple passing in pairs), low-intensity passing drills (≤30% of maximal effort), and dynamic upper-body and trunk mobility exercises. These activities are specifically selected because they do not target the hamstrings, impose no significant neuromuscular overload, and provide no performance-enhancing stimulus that could bias subsequent assessments. This approach ensures equal total active time across all groups and prevents a potential cooling-down effect before assessments. Following this controlled activity period, participants in the control group will resume their standard team-based soccer training sessions according to their regular schedule.

### 2.7 Measurements

#### 2.7.1 Basic information

The basic information to be collected includes age, standing height, leg length, body mass, body mass index, body fat percentage, experience, leg dominance, and additional weekly soccer training.

#### 2.7.2 Primary outcomes

##### 2.7.2.1 Hamstring shortness and knee range of motion

Using the active knee extension test [Intraclass Correlation Coefficient (ICC) = 0.98–0.99] (49), hamstring shortness via knee extension ROM will be assessed. The dominant limb, determined by having the participant kick a ball, will be designated as the test leg (50). The participant will be positioned comfortably supine. The test leg will be flexed to 90 degrees and stabilized with a strap to prevent pelvic movement; a towel will be placed under the spine to maintain spinal alignment. The contralateral leg will remain straight and be stabilized with a strap for accurate measurement. Anatomical markers will be placed on the greater trochanter (hip), external femoral condyle (knee), and lateral malleolus (ankle). Participants will actively extend their knees until they experience stretching discomfort. Images captured at a distance of 5 meters will be analyzed using Kinovea software version 0.9.5, a validated tool for ROM measurement (51–53). A cut-off value of more than 33 degrees will be used to indicate hamstring shortness (41).

##### 2.7.2.2 Muscle strength

For maximum strength assessment, a portable handheld dynamometer (HHD) (Lafayette Manual Muscle Testing System, Lafayette, IN, USA) will be utilized. Prior to testing, participants will receive detailed instructions. During each assessment, the participant's leg will be positioned at the starting point, and the participant will be instructed to exert maximum force against the HHD. Hamstring strength will be evaluated through both isometric and eccentric contractions, whereas quadriceps strength will be measured exclusively during isometric contractions. For isometric testing, the examiner will stabilize the limb by positioning the HHD perpendicular to the limb and maintaining a firm hold. Participants will progressively increase their effort over a three-second interval to reach maximum strength. Specifically, for hamstring isometric testing, the limb will be positioned with the knee flexed at 30 degrees, with the HHD placed 2 cm above the malleoli. For quadriceps isometric testing, the knee will be flexed to 60 degrees, ensuring contact between both popliteal fossae and the testing surface. In the eccentric hamstring assessment (54), the procedure closely mirrors the isometric test, except that participants are informed that the examiner will exert a downward pull on the calf for 3 s. The limb will be positioned with the knee flexed to 60 degrees, as verified with a universal goniometer. Each test will be performed twice per leg, with the maximum force recorded for subsequent analysis. Additionally, the ratios of isometric to eccentric hamstring strength will be computed (54). The ICC for this test is reported as 0.87 (55). Eccentric hamstring testing was included due to its established link to injury risk (54). Quadriceps is tested isometrically only, as this is standard for the H:Q ratio, and is highly reliable (ICC = 0.886) (54), and eccentric quadriceps weakness has received relatively little consideration in the literature (56), with no strong evidence linking it specifically to hamstring injury risk.

#### 2.7.3 Secondary outcomes

##### 2.7.3.1 Hamstring flexibility

The active straight-leg raise test will be used to assess hamstring flexibility (57). The participant will be positioned supine on a bed with the legs extended. The non-test limb will be immobilized using non-elastic straps to prevent compensatory movements. Markers will be affixed to the lateral epicondyle of the femur and the lateral malleolus of the ankle. The participant will then be instructed to actively elevate their dominant leg to its maximum height within two seconds, ensuring the knee of the test limb is fully extended. Upon reaching the maximum active ROM, the leg will be held in this position for one second, followed by a controlled return to the starting position within two seconds. Video recordings capturing the entire movement will be analyzed using Kinovea software. This procedure will be performed three times, and the maximum hip flexion angle will be recorded across trials. The reliability of this assessment, as evaluated by the ICC, is reported as 0.99 (5, 47, 57).

##### 2.7.3.2 Muscle power

The countermovement jump (CMJ) is primarily used to measure an athlete's explosive lower-body power (58). This test will begin with the participant standing with their hands on their hips. The participant will then perform a rapid squat to approximately 90 degrees of knee flexion, immediately followed by a maximal vertical jump. Participants will be instructed to land with their knees straight to improve jump-height accuracy. Jump height will be calculated using the My Jump Lab (My Jump 3) app (59) on a Samsung Galaxy S22 Ultra, by manually selecting take-off and landing frames from a video recording and applying the processes. The examiner assumes a prone position on the floor, with the mobile phone camera positioned 1.5 meters away at ground level, aiming at the participant's feet. Using the My Jump Lab application and the two specified formulas (Formulas 1, 2), the jump time, jump height, and peak power will be calculated. The ICC values for this assessment have reached up to 0.97 (60).

$$JH(cm)=t^{2}\times 1.22625,$$ (1)

$$Peak\;power\;(W)=61.9\times JH\;(cm)+36.0\times participant^{\prime }s\;body\;mass\;(kg)+1.822$$ (2)

##### 2.7.3.3 Sprint performance

The 20-meter sprint performance will be assessed utilizing the MySprint application. The procedure involves recording participants’ sprints using an Apple iPhone 13 running iOS 18. The camera will be mounted on a tripod at a frontal level, positioned 18 meters from the track. Two observers will manually identify the start frame (corresponding to the lift of the right big toe) and the end frame. The application will then automatically calculate the sprint time, with its validity confirmed through standard error of estimate values ranging from 0.007 to 0.015 s and high correlation coefficients between 0.989 and 0.999 (61, 62).

##### 2.7.3.4 Balance

Dynamic balance will be evaluated using the Y-balance test (YBT), based on performance metrics in the anterior, posteromedial, and posterolateral reach directions. In individuals with hamstring shortness, the YBT could be considered to assess potential balance deficits. Although hamstring tightness primarily affects mobility, YBT may reveal balance impairments that could benefit from interventions such as hamstring stretching (63, 64). First, each participant's lower limb length will be determined by measuring the distance from the anterosuperior iliac spine to the distal tibial malleolus with a 2-meter Lufkin tape measure. Then, participants will identify their dominant leg by indicating which leg they typically use to strike a ball (50). Subsequently, the YBT Kit will be used with participants performing the test barefoot. They will stand on the evaluated leg and reach with their free limb in the anterior, posteromedial, and posterolateral directions, following the YBT lower quarter protocol (65). Participants will keep their hands on their waist during the test. A trial will be considered invalid and repeated if the player releases their hands, moves their supporting foot, touches the ground with their free foot, loses balance, or fails to hold the starting position for one second after returning to center. Before the actual assessment, players will perform three familiarization trials, and the maximum reach distance in each direction will be recorded. The YBT demonstrated good inter-rater reliability with median ICC values of 0.88 (range: 0.83–0.96), 0.87 (range: 0.80–1.00), and 0.88 (range: 0.73–1.00) and intra-rater reliability with median ICC values of 0.88 (range: 0.84–0.93), 0.88 (range: 0.85–0.94), and 0.90 (range: 0.68–0.94) for the anterior, posteromedial, and posterolateral directions, respectively (66). The Composite Reach score is calculated using Formula 3.

Compositereachscore=(Reachdistances(cm))/(3×Limblength(cm))×100 (3)

##### 2.7.3.5 Proprioception

Joint position sense (JPS), a component of proprioception, is the ability to perceive and reproduce a joint angle without visual input (67). The dominant knee's JPS will be evaluated with the participant seated and wearing a blindfold. The knee will be passively moved from 90 degrees of flexion to a target angle of 45 degrees, as measured with a goniometer. The participant will then actively hold this position for five seconds and reposition to the target three times. Testing will occur at the same time of day for each participant. A video camera will record each session, capturing the movement of markers placed on specific anatomical landmarks: the fibula head, lateral malleolus, midpoint between the greater trochanter and the lateral epicondyle, and the lateral epicondyle (68). The recorded videos will be analyzed using Kinovea software, which demonstrates good to excellent reliability for knee joint measurements (ICC > 0.85 for intra-rater and > 0.90 for inter-rater reliability) with a disagreement of ±5° and ±2.5°, respectively (52). Consequently, the error score will be the absolute angular error, calculated as the difference between the target and achieved ROM (69).

### 2.8 Data collection and management

The data collection team comprises four PhD students specializing in Sports Science. Data collectors must complete standardized training and procedures for all tests. Table 1 outlines five measurement time points: baseline (t_0_), immediate post-first session measurement (t_1_), the intervention period spanning six weeks of training (t_2_), the chronic effect measurement taken six weeks after training (t_3_), and the follow-up assessment conducted three weeks after detraining (t_4_). Post-collection, participants’ identities will be anonymized using initials and numerical codes, and handwritten records will be digitized. Data will be independently entered into Microsoft 365 Excel (version 16.0, Build 2510) by two data collectors. Incomplete data entries will be recorded as unknown, missing, or not applicable. Following verification and cross-checking, all data will be stored on a password-protected computer accessible only to principal investigators, data consultants, and authorized data-entry personnel.

To ensure strict adherence to the intervention protocols and to monitor attendance, all training sessions (for three groups) will be directly monitored by four PhD students specializing in Sports Science at the participants’ regular training facilities. Attendance will be recorded for every session. During the detraining period, training load (duration and intensity) will be documented using coach logs and self-reported diaries. Intensity will be quantified using the session rating of perceived exertion (s-RPE) method (CR-10 Borg scale), with load calculated as RPE × session duration (minutes) (70).

### 2.9 Biological specimens

Not applicable for this study.

### 2.10 Data analysis

Data will be analyzed utilizing IBM SPSS Statistics (IBM Corp., 2021, Version 27, Armonk, NY) or GraphPad Prism 10 (GraphPad Software, Boston, MA, USA). Descriptive statistics, including means, standard deviations, and 95% confidence intervals, will be computed for all dependent variables at four distinct time points: baseline (pre-test), immediately following the initial session (acute effect), post-test after six weeks (chronic effect), and post-intervention follow-up after three weeks of detraining. The normality of the data distribution will be assessed using the Lilliefors test before applying parametric tests. A linear mixed-effects model with restricted maximum likelihood estimation will be used to analyze the effects of the interventions on the outcome measures. The model will include Intervention Group as the fixed between-subjects factor (three levels: Dynamic Stretching, Tissue Flossing, and Control), Time as the fixed within-subjects factor (four levels: Baseline, Acute, Chronic, and Post-detraining), and the Group × Time interaction. Participants will be included as a random effect to account for repeated measurements. This approach handles missing data under the Missing at Random assumption without requiring imputation and is robust to different dropout patterns. In cases of significant interaction effects, *post-hoc* analyses with Bonferroni adjustments will be conducted to delineate specific differences between groups at each time point. Effect sizes for the main effects and interactions will be reported as partial eta-squared (*η*p^2^). To quantify the magnitude of pairwise differences identified in *post-hoc* analyses, Cohen's d (d) will also be calculated and reported. Statistical significance will be determined at a *p*-value threshold of less than 0.05. With two primary outcomes (hamstring ROM and strength), correction for multiple comparisons (e.g., Bonferroni) will be applied only to these two outcomes. No correction for multiple comparisons will be applied to the secondary outcomes.

### 2.11 Oversight and monitoring

Trial oversight will be managed by the Principal Investigator (Hassan Daneshmandi), with a steering committee of co-investigators providing scientific guidance. Intervention fidelity will be ensured through standardized training and direct supervision of all sessions by the research team. Data integrity will be maintained through a dedicated monitoring protocol that involves double-entry and secure storage. Given the minimal-risk nature of the exercise interventions, formal independent data monitoring for efficacy is not planned; instead, the Principal Investigator will continuously monitor recruitment, adherence, and safety, reviewing any adverse events or protocol deviations to ensure participant safety and study validity.

### 2.12 Protocol amendments

If the research protocol requires modifications that could impact the implementation of the research, participant benefits, or safety considerations, such modifications must undergo formal revision. Such changes shall be subject to approval by the University of Guilan's Research Ethics Committees. The study protocol does not specify a particular stopping rule.

### 2.13 Quality control

To ensure scientific rigor and regulatory compliance, the protocol, initially approved by the Department of Sports Injury and Corrective Exercise at the University of Guilan, was subsequently reviewed multiple times by orthopedic and clinical research experts. Before starting the trial, all personnel must be trained on standard operating procedures to understand trial-specific protocols. During the trial, the team will hold weekly data safety meetings to review progress, clinical relevance, and adverse events. Meanwhile, trial monitors will conduct regular site visits to verify compliance with the guidelines.

## 3 Discussion

This protocol outlines a rigorous randomized controlled trial designed to elucidate the acute, chronic, and detraining effects of DS and TF on a comprehensive set of functional outcomes in adolescent soccer players with hamstring shortness. The rationale for this investigation is grounded in the high prevalence and detrimental impact of hamstring tightness in soccer, alongside the widespread but inadequately evidence-based use of these interventions in athletic settings (9, 10). While both modalities are employed to enhance flexibility and performance, a direct comparison of their longitudinal and residual effects on muscle function, sprinting, balance, and proprioception is lacking (4, 28). Conducting this study will therefore provide essential, granular evidence to guide practitioners in selecting and timing these interventions within athletic training and rehabilitation cycles.

To highlight the novelty of this randomized controlled trial, it is essential to distinguish its design from existing research. Previous studies have predominantly investigated the acute effects of DS and TF on isolated outcomes, such as flexibility or muscle activity (48, 71), or have examined TF in clinical populations (72) or for post-exercise recovery (73). In contrast, the present protocol is designed to directly compare DS and TF within the same athletic population across multiple time frames. Its novelty lies in the concurrent evaluation of acute, 6-week (chronic), and 3-week (detraining) effects on a comprehensive battery of functional and neuromuscular outcomes in adolescent soccer players with diagnosed hamstring shortness. In this regard, previous studies encourage exploring the long-term benefits of TF, highlighting its potential for sustained improvements beyond acute effects (4, 28). To the author's knowledge, no previous study has integrated this longitudinal, multi-domain comparison to inform both the efficacy and persistence of adaptations induced by these two distinct interventions.

The design of this trial incorporates several methodological strengths to generate robust evidence. The use of a three-arm parallel design with an active control group accounts for routine training effects, thereby facilitating the clear delineation of the specific impacts of the interventions. The assessment timeline, capturing acute, chronic, and detraining effects, represents a significant innovation, providing insights not only into the efficacy but also into the persistence of adaptations, both of which are essential for the development of training periodization strategies. Additionally, the comprehensive battery of outcome measures extends beyond basic flexibility assessments to include functional performance metrics (e.g., sprint and jump), neuromuscular control variables (e.g., balance and proprioception), and strength ratios. This approach provides a comprehensive assessment of how these interventions impact athletic capacity.

The successful execution of this study involves several practical considerations. First, recruiting and retaining adolescent athletes over a 10-week period (including baseline, 6-week intervention, and 3-week detraining phases) is challenging. Participant adherence may be influenced by academic schedules, competitive seasons, and motivation. To mitigate this, we will implement clear communication, flexible scheduling where possible, and maintain regular contact with participants and their clubs. The use of a control group that continues routine soccer training, with the option to receive the intervention post-study, is designed to aid retention while maintaining ethical standards. Second, ensuring the standardized application of the interventions is critical. While the DS protocol is inherently active and participant-driven, the TF application requires careful technique from the researchers to ensure consistent pressure (50% tension, 50% overlap) and positioning. To address this, all personnel administering the flossing intervention will undergo standardized training and periodic fidelity checks. Third, to control for diurnal variation, all outcome assessments will be conducted at the same time of day (± 1 h) for each participant across all four measurement time points. Fourth, the reliance on smartphone applications (My Jump, MySprint) and video analysis software (Kinovea) for primary outcome measures is not only cost-effective but also reflects their growing integration into contemporary sports practice, enhancing the ecological validity and practical relevance of the findings. The selected tools have demonstrated strong validity and high reliability in peer-reviewed research for assessing jump performance (59, 60) and sprint metrics (61, 62). Nevertheless, their use introduces specific operational considerations. Consistent device placement, lighting conditions, and standardized operator training are paramount to ensure data reliability. Our protocol specifies distances and angles for the camera setup, and all assessors will be trained to a common standard to mitigate these potential sources of error.

## 4 Limitations of the study protocol

Nevertheless, some issues and limitations are inherent in the design. First, the study focuses on a specific population: adolescent male amateur soccer players with diagnosed hamstring shortness. Therefore, the results may not be directly generalizable to female athletes, older populations, elite players, or individuals without hamstring tightness. Known sex-based differences in flexibility (74), hamstring injury epidemiology (5), and hormonal influences on connective tissue compliance (75) should be considered when interpreting our findings. Second, while the 3-week detraining period provides initial insights into the retention of effects, a longer follow-up would be necessary to fully characterize washout dynamics and inform off-season or injury-prevention programming. Third, the physiological mechanisms underlying any observed differences, such as changes in fascial sliding, pain tolerance, neuromuscular activation, or perfusion, will not be directly measured. While we hypothesize that the distinct mechanisms proposed for each technique (neuromuscular vs. mechanical/vascular) may be at play, the study is designed to compare functional outcomes rather than elucidate these mechanisms. Fourth, HHD for strength assessment depends on the examiner's strength and stability, particularly for larger, stronger adolescent athletes. Although all examiners will receive standardized training and extensive supervised practice to minimize this effect, it remains a potential limitation. Fifth, the cut-off value for hamstring shortness (>33° on the active knee extension test) was derived from a young adult population (18–24 years) (41). To our knowledge, no adolescent-specific cut-off values have been established in the literature. While the active knee extension test has demonstrated good reliability in children aged 10–13 years (76), the applicability of this specific cut-off to our adolescent population (14–17 years) is a limitation. Finally, as with many exercise interventions, blinding participants and therapists is not feasible, which may introduce expectancy bias. We aim to mitigate this by using objective, instrument-based outcome measures where possible and by standardizing all instructions.

## 5 Conclusion

In conclusion, this rigorously designed trial protocol will provide a robust comparison of two commonly used intervention strategies for hamstring shortness. By evaluating their effects across multiple timeframes and on a range of functional and neuromuscular outcomes, the study aims to move beyond a simple which is better dichotomy. Instead, it seeks to establish an evidence-based framework for practitioners to match the specific temporal (acute warm-up vs. chronic adaptation) and functional (flexibility vs. power vs. control) goals of their athletes with the most appropriate intervention. The findings are expected to directly inform warm-up routines, in-season maintenance programs, and targeted intervention strategies for adolescent soccer players, with potential relevance to other field sports as well.
